# Entwicklung der Analytik im Neugeborenen-Screening – Von der Guthrie-Karte zur Genetik

**DOI:** 10.1007/s00103-023-03774-5

**Published:** 2023-10-12

**Authors:** Nils Janzen, Johannes Sander

**Affiliations:** 1 Screening-Labor Hannover, Hannover, Niedersachsen Deutschland; 2 Institut für Klinische Chemie, Medizinische Hochschule Hannover, Hannover, Niedersachen Deutschland; 3 Abteilung Klinisches Labor, Kinder- und Jugendkrankenhaus Auf der BULT, Hannover, Niedersachen Deutschland; 4 Institut für Medizinische Mikrobiologie und Krankenhaushygiene, Medizinische Hochschule Hannover, Hannover, Niedersachen Deutschland; 5 Institut für Klinische Chemie, Medizinische Hochschule Hannover, Carl-Neuberg-Str. 1, 30625 Hannover, Deutschland

**Keywords:** Guthrie-Test, Früherkennungsuntersuchung, Gendiagnostik, Tandem-Massenspektrometrie, Angeborene Stoffwechselkrankheiten, Guthrie test, Healthcare screening, Genetic diagnostics, Tandem mass spectrometry, Inborn errors of metabolism

## Abstract

Seit mehr als 5 Jahrzehnten wird allen Neugeborenen in Deutschland eine Vorsorgeuntersuchung zur Früherkennung angeborener behandelbarer Krankheiten angeboten. Seit Beginn sind so etwa 35 Mio. Kinder untersucht worden.

Anfangs ging es nur um die Früherkennung der Phenylketonurie, die ohne frühzeitige Behandlung zu nicht mehr korrigierbarer geistiger Behinderung führt. Der bakteriologische Guthrie-Test erlaubte den Nachweis erhöhter Konzentrationen von Phenylalanin. Die heute eingesetzten Methoden sind das Ergebnis einer über Jahrzehnte verlaufenden Entwicklung. Hinzugekommen sind Tests zur Bestimmung von Enzymaktivitäten, Immunoassays zur Früherkennung wichtiger hormoneller Störungen wie der angeborenen Schilddrüsenunterfunktion sowie Hochdruck-Flüssigkeits-Chromatografie zur Identifizierung pathologischer Hämoglobine. Die sehr anspruchsvolle Tandem-Massenspektrometrie ermöglicht die gleichzeitige Erfassung von Aminosäuren und Derivaten organischer Säuren und Fettsäuren. Auch Steroide können damit identifiziert werden. Die Spezifität lässt sich durch Kombination mit chromatografischer Vortrennung noch erhöhen. In den letzten Jahren wurden die chemisch-analytischen Untersuchungen ergänzt durch gendiagnostische Verfahren, wie beispielsweise quantitative oder qualitative Polymerasekettenreaktion (PCR).

Der Stand der Labortechnik ist keineswegs endgültig. Sowohl die klassische Analytik als auch besonders die genetischen Verfahren stehen vor einer weiteren rasanten Entwicklung. Während die Ausweitung des Screenings auch eine Folge der technischen Entwicklung ist, hängt die Einbeziehung weiterer angeborener Erkrankungen grundsätzlich von einer jeweiligen Therapie ab. Aber gerade hier werden gegenwärtig viele Neuerungen erprobt. Im Vordergrund des Interesses steht dabei die Gentherapie.

## Abstract (en)

For more than five decades, all newborns in Germany have been offered a screening examination for the early detection of congenital treatable diseases. Since its inception, about 35 million children have been screened in this way.

Originally, screening exams only included early detection of phenylketonuria, which, without timely treatment, would lead to mental retardation that could no longer be corrected. The bacteriological Guthrie test allowed the detection of elevated concentrations of phenylalanine. The methods used today are the result of decades of development. They have been expanded to include tests to determine enzyme activities, immunoassays for the early detection of important hormonal disorders such as congenital hypothyroidism, and high-pressure liquid chromatography for the diagnosis of pathologic hemoglobins. The very sophisticated tandem mass spectrometry enables the simultaneous detection of amino acid and fatty acid compounds. Steroids can also be identified. The specificity can be further increased by combining tandem mass spectrometry with chromatographic pre-separation. In recent years, chemical-analytical analyses have been supplemented by genetic diagnostic methods such as quantitative or qualitative polymerase chain reaction (PCR).

The current state of laboratory technology is by no means final. Both classical analytics and especially genetic methods are facing further rapid development. Although the expansion of screening is also a consequence of technical development, the inclusion of further congenital diseases is fundamentally dependent on the given therapy. But it is precisely here that many innovations are currently being investigated. Gene therapy is at the forefront of interest.

## Einleitung

Das Untersuchungsprogramm zur Früherkennung angeborener Krankheiten, die gleich nach der Geburt symptomarm oder symptomlos sind, in der Folge aber schwere Gesundheitsschäden verursachen, blickt auf eine Geschichte von mehr als 5 Jahrzehnten zurück. Von Anfang an war es als „Siebtest“ (Sieb, engl.: „screen“) konzipiert. Ziel war es, grundsätzlich alle Neugeborenen einzubeziehen, um die wenigen Positiven „auszusieben“.

Zunächst ging es nur um die Früherkennung der Phenylketonurie (PKU), die zu dieser Zeit behandelbar wurde. Sie beruht auf einem autosomal rezessiv vererbten Defekt der Phenylalaninhydroxylase. Phenylalanin reichert sich an und wird über andere Stoffwechselwege in z. T. toxische Produkte umgesetzt. Der Widerstand gegen die Einführung einer alle Neugeborenen umfassenden Untersuchung war zunächst groß bei einer vermuteten Häufigkeit der gesuchten Erkrankung von nur 1:10.000 bis 1:20.000. Überzeugt hat schließlich die Erkenntnis, dass sich PKU-Patienten nach frühzeitiger Diagnose und adäquater lebenslanger Therapie weitestgehend unauffällig entwickeln. PKU-Patienten machten vor Einführung des Neugeborenen-Screenings einen erheblichen Anteil der in Heil- und Pflegeanstalten versorgten Personen aus.

Als vor ca. 60 Jahren die Idee einer generellen Vorsorgeuntersuchung aller Neugeborenen auf eine schwerwiegende angeborene Stoffwechselstörung in die Tat umgesetzt werden sollte, gab es zunächst nicht nur keine geeigneten analytischen Verfahren, sondern es fehlte auch eine Möglichkeit, die winzigen bei Neugeborenen zu gewinnenden Proben aus Kliniken, Arztpraxen und von niedergelassenen Hebammen sicher zu geeigneten Laboratorien zu transportieren. Es war wohl Robert Guthries Idee, Bluttropfen auf Filterpapierkarten aufzutragen und dann in getrocknetem Zustand durch übliche Briefpost an zentrale Screening-Labore zu verschicken. Anfangs hatte man versucht, die Neugeborenen nicht mit einer Blutentnahme zu belasten. Die Probenlogistik hat sich sehr bewährt und es besteht seit 60 Jahren kein Anlass, sie zu verändern. Die Idee, stattdessen Nabelschnurblut oder Urin zu verwenden, hat sich aber aus verschiedenen Gründen nicht durchgesetzt. 

Die Technik der Laboranalysen hat dagegen eine revolutionäre Entwicklung genommen. Der Fortschritt der Untersuchungstechnik hat auch eine schrittweise Ausweitung des Screening-Programms auf den heutigen Stand [1] erlaubt.

Die rasante, nicht lineare Entwicklung der Laboranalytik insgesamt und im Neugeborenen-Screening soll in dieser Übersicht deutlich werden. Die derzeitig eingesetzten Verfahren werden beschrieben und ihre Entwicklungsgeschichte kurz skizziert. Wegen ihrer historischen Bedeutung wird auch kurz auf die nicht mehr üblichen bakteriologischen Testmethoden eingegangen. Die klassischen Analyseverfahren einschließlich der schon lange bewährten Hormontests werden als noch heute sehr wichtige Methoden angesprochen. Besondere Beachtung finden die neuen Verfahren der Tandem-Massenspektrometrie und der genetischen Analyse. Die Arbeit zeigt auch auf, dass die Entwicklung des Screenings keineswegs abgeschlossen ist und sich auch an den Fortschritten, die in anderen Industrieländern erzielt werden, messen lassen muss.

## Bakteriologische Tests

Eine erste „Welle“ der Neugeborenen-Vorsorgeuntersuchung umfasste die Einführung und Optimierung des bakteriologischen Tests zum Nachweis erhöhter Konzentrationen von Phenylalanin, der von Guthrie und Susi [2] entwickelt wurde. Das Testprinzip ist einfach: Aus den blutgetränkten Arealen der Karten ausgestanzte Scheibchen werden auf bakteriologische Agar-Nährböden aufgelegt, die eine unsichtbare Suspension von Bacillus-subtilis-Sporen und einen Hemmstoff enthalten, der das Auskeimen der Sporen verhindert. Aus den Stanzlingen der Karten diffundiert die gesuchte Substanz, z. B. Phenylalanin, radial in den feuchten Nährboden, wobei ein Konzentrationsgradient entsteht. Phenylalanin hebt lokal die Wirkung des Hemmstoffs auf. Je höher die Konzentration der Aminosäure ist, desto größer werden die Wachstumshöfe, die visuell beurteilt werden.

Der Erfolg der ersten Guthrie-Tests in den USA hat in kurzer Zeit zur Übernahme in alle Industriestaaten geführt. In Deutschland begannen erste Screening-Untersuchungen Mitte der 1960er-Jahre. Die Idee des bakteriologischen Tests wurde ausgebaut und zur Früherkennung weiterer Aminoazidopathien und der Galaktosämie eingesetzt.

Der Guthrie-Test ist zwar leicht und kostengünstig durchzuführen, wird als bakteriologischer Test aber durch Antibiotika gestört. Eine antibakterielle Therapie des Kindes oder der Mutter können den Test beeinflussen. Es wurde über falsch-negative Ergebnisse berichtet, die zu schweren geistigen Behinderungen führten. Der Test erfasst außerdem nur die gesuchte Aminosäure allein. Der Blutspiegel des Phenylalanins wird aber nicht nur durch die Aktivität der Phenylalaninhydroxylase, sondern auch durch Lebensalter in Tagen, durch Ernährung, Infusionen und weitere Faktoren beeinflusst. Eine sicherere Diagnose würde sich ergeben, wenn der Test nicht nur das Ausgangsprodukt der Reaktion, Phenylalanin (Phe), sondern auch das Reaktionsprodukt, Tyrosin (Tyr), quantifizieren würde. An dem Konzentrationsverhältnis beider Aminosäuren lässt sich die angeborene Enzymstörung zuverlässig demonstrieren. Die Berechnung des Phe/Tyr-Quotienten ist heute Bestandteil der moderneren PKU-Nachweise.

Mit der allgemeinen Entwicklung der Analysentechnik erlebten auch die Screening-Labore eine ständige Anpassung der Methodik. Der bakteriologische Guthrie-Test, der den Siegeszug der biochemischen Neugeborenen-Vorsorgeuntersuchung ermöglicht hatte, ist in den 1970er-Jahren zunehmend durch präzisere und schnellere Verfahren abgelöst worden. Diese neueren photometrischen oder chromatografischen Verfahren werden nicht durch Antibiotika beeinflusst.

## Klassische chemische Analyseverfahren

In nicht mikrobiologisch arbeitenden Institutionen lag es von vornherein nahe, Screening-Programme aufzubauen, die auf klassischen, gut etablierten klinisch-chemischen Verfahren fußten. Eine Möglichkeit bestand darin, Phenylalanin und/oder andere Aminosäuren mithilfe der Dünnschichtchromatografie [3, 4] zu bestimmen. Proben können dabei in größerer Zahl parallel verarbeitet werden.

Sehr elegant ist die fluorometrische Messung von Phenylalanin [5]. Die Fluorometrie ist preisgünstig und lässt sich gut automatisieren. Allerdings wird, wie im Guthrie-Test, in jedem Analysengang nur eine Aminosäure, z. B. Phenylalanin, gemessen. Für die Früherkennung von Aminoazidopathien wie PKU oder Ahornsirupkrankheit („maple syrup urine disease“, MSUD) wurden diese Techniken nach Einführung der Tandem-Massenspektrometrie (TMS) aufgegeben. Die für die Frühdiagnostik der Galaktosämie notwendige Messung der Konzentration von freier Galaktose, Galaktose-1-Phosphat oder Gesamtgalaktose erfolgt auch heute noch durch einen photometrischen oder fluorometrischen Test.

Zu den klassischen chemischen Verfahren gehören auch Tests, bei denen die Enzymaktivitäten im Blut der Probanden gemessen werden. Die Bestimmung der Enzymaktivität der Galaktose-1-Phosphat-Uridyltransferase und der Biotinidase wurden 1975 bzw. 1985 in das deutsche Neugeborenen-Screening aufgenommen. [6].

## Hormonbestimmungen

### Konnatale Hypothyreose

Einige wichtige angeborene Krankheiten betreffen den Hormonhaushalt. Die konnatale Hypothyreose ist, wenn man die Zahl der behandlungsbedürftigen Kinder als Maßstab nimmt, wohl die wichtigste. Jeweils eines von ca. 3000 Neugeborenen ist betroffen. Ein Mangel an Thyroxin (T4) hat einige Neugeborene bereits intrauterin so stark geschädigt, dass eine visuelle Verdachtsdiagnose möglich wäre, aber in der ganz überwiegenden Mehrzahl der Fälle erscheinen hypothyreote Kinder in den ersten Lebenstagen klinisch unauffällig. Ein Neugeborenen-Screening auf konnatale Hypothyreose wurde möglich, nachdem geeignete Radioimmunoassays zur Bestimmung von T4 und Thyreoidea stimulierendem Hormon (TSH) entwickelt waren [7–9].

In Deutschland wurde die Hypothyreose 1978 als Zielkrankheit in das Neugeborenen-Screening aufgenommen. Als technische Varianten wurden Immunoassays entwickelt, die ohne Verwendung radioaktiver Indikatoren mit farbbildenden Enzymreaktionen oder Fluoreszenzsignalen arbeiten [10].

Die primäre Hypothyreose tritt sporadisch auf. Es handelt sich nicht um eine vererbte Krankheit. Prinzipiell kann ein Hypothyreose-Screening auf der Bestimmung des Schilddrüsenhormons T4 in freier oder gebundener Form aufbauen. Da die Geburt und die Anpassung an das extrauterine Leben mit erheblichem Stress verbunden sind, führt die Messung von T4 allerdings zu deutlich schwankenden Werten [11].

Einen weniger volatilen Parameter stellt TSH dar. Der TSH-Screeningtest findet eine primäre konnatale Hypothyreose bei Reifgeborenen mit sehr großer Sicherheit. Mütterliche Faktoren wie die maternale Hypo- und ebenso die Hyperthyreose können jedoch das Ergebnis beeinflussen. Antithyreoidale IgG-Antikörper werden diaplazentar übertragen. Jodmangel und Jodüberdosierung, z. B. durch jodhaltige Desinfektionsmittel oder Medikamente, können den Test stark verfälschen [12]. Sehr großen Einfluss hat das Gestationsalter des Neugeborenen. Das noch nicht voll etablierte Zusammenspiel von Hypophyse und Schilddrüse ist bei sehr unreifen Frühgeborenen Ursache vieler falsch-positiver und -negativer Resultate.

Die „Kinder-Richtlinie“^1^ [13] unterscheidet nicht zwischen primärer und sekundärer Hypothyreose und schreibt das Testverfahren nicht explizit vor. Demnach wären Bestimmungen des T4 ebenso zulässig wie die Messung des TSH-Wertes oder eine Kombination von beiden. Letztgenanntes ist Grundlage des Hypothyreose-Screenings in einigen ausländischen Programmen, ist aber mit einer erhöhten Zahl kontrollbedürftiger Ergebnisse verbunden [14].

### Adrenogenitales Syndrom (AGS)

Die Früherkennung des AGS (seit 2002 Teil des Neugeborenen-Screenings in Deutschland) ist besonders wichtig, weil die klassische Form schon innerhalb weniger Tage nach Geburt zu einer lebensbedrohlichen Hyponatriämie führen kann. Eine wirksame Therapie ist gegeben, muss aber sehr früh einsetzen; jeder Tag zählt.

Der kongenitalen Nebennierenhyperplasie (CAH) liegt in über 90 % der Fälle ein Defekt der 21-Hydroxylase, eines von mehreren Enzymen in der Synthesekaskade der Steroidhormone, zugrunde, der autosomal rezessiv vererbt wird. Der 21-Hydroxylase-Mangel verhindert die Umsetzung von u. a. 17-Hydroxyprogesteron (17-OHP) in 11-Desoxycortisol. Bei Mädchen beobachtet man eine unterschiedlich stark ausgeprägte Virilisierung, während neugeborene Jungen zunächst klinisch meist unauffällig sind. Diagnostischer Parameter für das Screening ist 17-OHP. Standardverfahren ist zurzeit noch die Bestimmung mithilfe eines Immunoassays, obwohl ein besseres Verfahren zur Verfügung steht [15, 16].

Die Höhe der 17-OHP-Konzentration hängt nicht nur von der Aktivität der 21-Hydroxylase ab, sondern wird, ähnlich wie bei der TSH-Bestimmung, von zahlreichen weiteren Faktoren beeinflusst. Der Geburtsstress spielt eine wichtige Rolle. Einen besonderen Einfluss hat auch das erreichte Gestationsalter. Das Zusammenspiel der verschiedenen an der Steroidsynthese beteiligten Enzyme macht im Verlauf des fetalen Wachstums einen Reifeprozess durch, der bei Frühgeborenen noch nicht abgeschlossen ist. Die Steuerung der Nebenniere über die Ausschüttung des adrenocorticotropen Hormons (ACTH) des Hypothalamus nimmt ihre volle Funktion erst mit zunehmendem Gestationsalter auf. Dementsprechend erhält man bei sehr unreifen Neonaten bei der 17-OHP-Bestimmung u. U. hohe und sehr hohe Konzentrationen, ohne dass ein genetischer Defekt vorliegt. Umgekehrt kann in dieser Gruppe der Neugeborenen der Konzentrationsanstieg trotz gestörter Funktion der 21-Hydroxylase noch fehlen.

Der Vorhersagewert, der sog. positiv prädiktive Wert (PPV), der immunometrischen 17-OHP-Bestimmung ist relativ gering, im Jahresbericht 2020 der Deutschen Gesellschaft für Neugeborenenscreening e. V. (DGNS; [17]) wird er mit 6,5 % angegeben. Erfolgte die Geburt vor der 32. Schwangerschaftswoche, so erreicht der PPV nur 1–3 %. Mit anderen Worten, mit diesem Test lässt sich ein AGS bei Frühgeborenen nicht unmittelbar postnatal sicher erkennen.

Weitere Probleme des 17-OHP-Screenings sind technischer Art. Die verschiedenen Steroide weisen viele gemeinsame Strukturelemente auf. Kreuzreaktionen des Immunoassays mit anderen Steroiden verfälschen deshalb die Ergebnisse. Gelegentlich kommen auch Kontaminationen der Blutprobe mit EDTA vor, die hohe Testergebnisse vortäuschen [18]. Will man die Vorsorgeuntersuchung auf AGS grundsätzlich verbessern, so ist dies durch parallele Bestimmung mehrerer Steroide möglich [16].

## Hochdruck-Flüssigkeits-Chromatografie (HPLC)

Zu den Zielkrankheiten des Screenings gehört auch die Sichelzellanämie, eine autosomal-rezessiv vererbte Hämoglobinanomalie. Bei homozygot Betroffenen findet man das veränderte Hämoglobin neben der fetalen Variante, während das adulte Hämoglobin A fehlt. Heterozygote sind gesund. Von klinischer Relevanz ist aber eine Compound-Heterozygotie, die auch als HbSC-Krankheit bezeichnet wird. Sie kommt in Westafrika gehäuft vor und wird in Deutschland bei Migranten aus dieser Region gefunden. Weitere Kombinationen sind möglich.

Zum Nachweis des Sichelzell-Hämoglobins HbS und weiterer Varianten ist in der „Kinder-Richtlinie“ der Einsatz der HPLC vorgesehen. Die vorgegebene Methodik lässt die pathologischen Hämoglobinvarianten sicher erkennen, ist als Screeningverfahren aber sehr aufwändig. Daher wird in einigen Laboren eine genetische Bestimmung im Hochdurchsatzverfahren angewandt, die durch eine *Second-tier*-Methode (HPLC, Elektrophorese, TMS) ergänzt wird [19].

## Tandem-Massenspektrometrie (TMS, MS/MS)

Mit der Einführung der TMS ergab sich erstmals die Möglichkeit, mehrere Erkrankungen mit einem einzigen Analysenverfahren gleichzeitig zu detektieren. Damit wurden die Voraussetzungen geschaffen, diese Erkrankungen mit unterschiedlicher Inzidenz und Therapierbarkeit frühzeitig zu behandeln. Mit dieser hochsensitiven und -spezifischen Analysenmethode werden Störungen im Aminosäuren- und Fettsäurestoffwechsel sowie des Transports und Abbaus von Fettsäuren erfasst. Sowohl die PKU als auch die MSUD können sehr gut mittels TMS detektiert werden. Auch die Organoazidurien, weitere Erkrankungen des Eiweißstoffwechsels, die zu einer Anhäufung organischer Säuren in Blut und Urin führen, sind gut nachweisbar.

Sowohl bei der MSUD als auch bei der Isovalerianazidurie (IVA) handelt es sich um eine Störung des Abbaus von Leucin. Die Glutarazidurie Typ I wird durch eine Störung des Abbaus der Aminosäuren Lysin, Hydroxylysin und Tryptophan verursacht. Folge ist eine Akkumulation von toxischen Abbauprodukten in allen Organen und Körperflüssigkeiten. Unbehandelt führen diese Erkrankungen zu schweren irreversiblen neurologischen Schädigungen.

Unter den Enzymdefekten des Fettsäurestoffwechsels ist der MCAD-Mangel (Medium-Chain-Acyl-CoA-Dehydrogenase) der häufigste (ca. 1:10.000). Die Häufigkeit ist höher, als vor Einführung des erweiterten Neugeborenen-Screenings bekannt war. Diese Erkrankung ist durch eine Akkumulation von mittellangkettigen Acylcarnitinen charakterisiert, die sich mittels TMS sehr gut nachweisen lassen. Weitaus seltener sind Störungen der Enzyme, die langkettige und sehr langkettige Fettsäuren abbauen, sowie Transporterstörungen. Die klinische Symptomatik ergibt sich einerseits durch die Unfähigkeit der Betroffenen, Fettsäuren als Energiequelle zu nutzen, und andererseits durch die toxischen Wirkungen der sich ansammelnden Stoffwechselzwischenprodukte. Ausgelöst durch katabole Stoffwechsellagen kann es zu lebensbedrohlichen Episoden von hypoketotisch-hypoglykämischem Koma kommen.

Massenspektrometrie beruht auf der Identifizierung elektrisch geladener Moleküle, die in einem elektromagnetischen Feld nach dem Verhältnis von Masse zu Ladung separiert und quantifiziert werden [20]. Für das Screening werden aus Trockenblut gewonnene Probenextrakte, die eine Vielzahl von Metaboliten enthalten, direkt oder nach Derivatisierung einer Ionenquelle zugeführt. Dort werden sie elektrisch geladen, bilden somit Ionen. Diese gelangen direkt (*Flow Injection*, FI-MS/MS) in Form eines Sprays in das Tandem-Massenspektrometer, welches aus 2 hintereinander geschalteten Massenspektrometern (Quadrupolen) sowie einer Kollisionszelle besteht.

Die im ersten Quadrupol aufgetrennten Ionen werden in der Kollisionszelle durch Kollision mit Argon oder Stickstoff in charakteristische Fragmente zerlegt und diese anschließend im zweiten Quadrupol aufgetrennt. Danach treffen sie auf einen Detektor, der unter Verwendung von spezifischen Standards die quantitative Analyse der gesuchten Metabolite ermöglicht. Zur Auswertung der Messungen werden nicht nur die Konzentrationen der Metabolite herangezogen, sondern auch charakteristische Quotienten und Summen. Für einige Erkrankungen sind diese Messergebnisse so spezifisch, dass sich die Verdachtsdiagnose aus dem Screening fast immer bestätigt.

Im Screening werden Aminosäuren und Carnitinester von Fett- und organischen Säuren parallel bestimmt. Die Analysenzeit je Probe beträgt weniger als 2 min. Ein wesentlicher Nachteil dieser Methode für die Praxis besteht darin, dass einige strukturell ähnliche Substanzen identische Masse‑/Ladungsverhältnisse (m/z) aufweisen und daher falsch-positive Ergebnisse liefern können. Die Kombination der TMS mit der Flüssigkeits-Chromatografie (LC-MS/MS) erlaubt die Auftrennung isobarer Moleküle mit identischen m/z und führt zu einer sensitiveren Bestimmung der jeweiligen Substrate [21]. Die LC-MS/MS wird bisher bevorzugt als Zweitmethode (*second Tier*) eingesetzt, um unsichere Ergebnisse des Primärscreenings, wie oben erwähnt, zu verifizieren. Dies erfordert in der Regel längere Analysenzeiten und mehr technisches Equipment.

Die LC-MS/MS könnte für eine ständig größer werdende Zahl angeborener Krankheiten als Primärverfahren eingesetzt werden, aber sie eignet sich nicht dazu, FI-MS/MS in den bisherigen Anwendungen vollständig zu ersetzen. Die Entwicklung eines LC-MS/MS-Screenings würde die Etablierung einer weiteren analytischen Methodenlinie bedeuten. Angesichts der Aussicht, dass viele weitere angeborene Erkrankungen in naher Zukunft therapierbar werden, sind entsprechende Überlegungen nicht verfrüht. International gibt es eine wachsende Zahl von Screening-Laboren, die diesen Weg bereits beschreiten [22].

Tandem-Massenspektrometer sind sehr komplexe, teure Instrumente. Reparaturen und tiefgreifende Geräteeinstellungen ohne Herstellersupport sind nur eingeschränkt möglich. Die Geräte sind zwar bedienerfreundlich, aber ohne ein gutes Verständnis der messtechnischen Abläufe und der komplexen Soft- und Hardware ist ein verantwortungsvoller Einsatz im Screening nicht möglich; speziell qualifiziertes Personal und ständige Weiterbildung sind erforderlich.

## Molekulargenetische Untersuchungen

### Cystische Fibrose (CF)

Zum 01.09.2016 wurde aufgrund eines Beschlusses des Gemeinsamen Bundesausschusses (G-BA) die Untersuchung auf Mukoviszidose (cystische Fibrose, CF) in das Neugeborenen-Screening aufgenommen [13]. Dies war der Start von molekulargenetischen Untersuchungen im Neugeborenen-Screening in Deutschland. Bereits zuvor unterlag das Screening dem Gendiagnostik-Gesetz [23]. Jedoch handelte es sich bei den Untersuchungen bis dahin um semiquantitative oder quantitative Bestimmungen von Enzymaktivitäten, Substraten und Hormonen. In der „Kinder-Richtlinie“ wird das CF-Screening in einem eigenen Kapitel getrennt vom erweiterten Neugeborenen-Screening geführt.

CF ist die häufigste lebensverkürzende autosomal-rezessiv vererbte Krankheit in der europäischen Bevölkerung. Mutationen des auf Chromosom 7 lokalisierten Gens für *Cystic Fibrosis Transmembrane Conductance Regulator* (CFTR) sind dafür verantwortlich. Mehr als 2000 Mutationen sind bekannt, F508del wird am häufigsten gefunden.

Die Untersuchung auf CF erfolgt in Deutschland nach einem Stufenplan: In Abhängigkeit von positiven Ergebnissen der immunologischen Nachweismethoden auf immunreaktives Trypsin (IRT) und auf Pankreatitis-assoziiertes Protein (PAP) ist nun grundsätzlich zur Bestätigung eine molekulargenetische Untersuchung auf die in Deutschland häufigsten 31 Mutationen des CFTR-Gens vorgesehen. Dies geschieht mithilfe einer Polymerasekettenreaktion (PCR) und der qualitativen Identifikation der Reaktionsprodukte. Die klinische Verifizierung erfolgt dann mit der Durchführung eines Schweißtests und oftmals auch zusätzlicher genetischer Methoden.

### Schwerer kombinierter Immundefekt (SCID)

Im Jahr 2021 folgte die Einführung weiterer gendiagnostischer Untersuchungen. Der schwere kombinierte Immundefekt (*SCID – Severe Combined Immuno Deficiency;* eine Gruppe von angeborenen Immundefekten; [24]) und die spinale Muskelatrophie (SMA; [25]) wurden Zielkrankheiten des Neugeborenen-Screenings. Bei den Untersuchungen handelt es sich jeweils um PCR-Methoden mit anschließender Detektion der vorliegenden monogenetischen Mutation. Beide Methoden weisen bekannte Veränderungen in bestimmten Regionen (Exomen) der kindlichen DNA nach.

Die dem SCID zugrunde liegende Störung verhindert die normale Entwicklung des Immunsystems. Bei Betroffenen sind Funktionsverluste der T‑ und der B‑Lymphozyten kombiniert. Der Screening-Test weist *T‑Cell-Receptor-Excision-Circles* (TREC) nach. Dies sind DNA-Fragmente, die während der Formierung des T‑Zell-Rezeptors entstehen. Letzteres ist ein Protein auf der Oberfläche von T‑Zellen, das der Unterscheidung körpereigener von körperfremden Antigenen dient. Da infektionsprophylaktische und -therapeutische Maßnahmen sehr frühzeitig erfolgen müssen, stellt der positive Nachweis des TREC-Mangels einen immunologischen Notfall dar.

Der Test kann auch durch andere Erkrankungen, (mütterliche) Medikation sowie Lymphozytopenien zu einem auffälligen, d. h. falsch-positiven Ergebnis führen, das dann weitere klinische Untersuchungen auslöst [26]. Heilung des Immundefektes ist möglich durch Transplantation hämatopoetischer Stammzellen.

### Spinale Muskelatrophie (SMA)

Die Mehrzahl der SMA-Betroffenen zeigt eine homozygote Deletion des SMN1-Gens auf Chromosom 5. Ein Mangel an *survival-motor-neuron*(SMN)-Protein und damit eine fortschreitende Zerstörung von Motorneuronen des Rückenmarks sind die Folge. Heterozygote Merkmalsträger, also Neugeborene mit Deletion des SMN1-Gens auf nur einem Allel, sind nicht krank und werden vom Test nicht erfasst. Letzteres gilt allerdings auch für Compound-Heterozygote, die auf einem Allel eine Deletion und auf dem anderen eine Punktmutation zeigen und erkranken. So werden durch das Screening nur ca. 95 % aller Fälle diagnostiziert.

Die Schwere des klinischen Bildes wird modifiziert durch das Vorhandensein eines zweiten Gens (SMN2-Gen). Dieses zweite Gen findet man in individuell unterschiedlicher Kopienzahl (1–6 Kopien). Das Vorhandensein von SMN2 erlaubt ebenfalls die Bildung von SMN-Protein, jedoch nur in begrenztem Ausmaß. Je höher die Kopienzahl des SMN2-Gens, desto mehr SMN-Protein wird gebildet und desto leichter ist der klinische Verlauf. Eine sofortige Bestimmung der Zahl der vorhandenen Kopien des SMN2-Gens noch aus der Erstprobe wäre möglich, ist aber nach der „Kinder-Richtlinie“ nicht vorgesehen. Dies soll vielmehr im Zusammenhang mit der klinischen Untersuchung in einem neuropädiatrischen Zentrum mit Erfahrung in der Betreuung von SMA-Patienten erfolgen.

## Künftige Entwicklung

Die über die Jahre erfolgte Ausweitung des Neugeborenen-Screenings wurde einerseits durch zunehmend ausdifferenzierte Möglichkeiten einer massengeeigneten Analytik geprägt. Die entscheidenden Impulse kamen aber aus der Klinik durch ständige Verbesserung der Therapie. Dies wird auch in Zukunft so sein. Standen im Anfang der Entwicklung Diät und später die Gabe von Hormonen und speziellen Medikamenten im Vordergrund, so treten gegenwärtig neue Therapiekonzepte hinzu. Fehlende Enzyme können u. U. durch Ersatzprodukte substituiert werden [27]. Ganz neue Horizonte eröffnen sich mit der Einführung genetischer Therapien [28–32]. Es besteht Hoffnung, dass zumindest einige der vielen bekannten Gendefekte dauerhaft geheilt werden können. Schon jetzt stehen verschiedene In-vitro- und In-vivo-Behandlungen zur Verfügung.

Das bestehende Neugeborenen-Screening hat sich als ein segensreiches Programm erwiesen. Rund 35 Mio. Kinder sind damit inzwischen in Deutschland untersucht worden. Verbesserungen sind dennoch möglich und eine Anpassung an den allgemeinen medizinischen Fortschritt, wie in der Vergangenheit, ist vorhersehbar.

### Verbesserungen

Zu erwartende Verbesserungen betreffen u. a. die in der „Kinder-Richtlinie“ angegebene Methodik. Einige derzeit vorgegebene Verfahren führen zu einer unnötig hohen Zahl von falsch-positiven Resultaten. Die Festlegung spezifischer Zweituntersuchungen (*second tier*) mit ergänzender Methodik kann Abhilfe schaffen.

So ist, wie erwähnt, der PPV einer 17-OHP-Bestimmung mit dem vorgegebenen immunometrischen Test vor allem bei Frühgeborenen sehr gering. Ein ergänzend mit LC-MS/MS erstelltes Steroidprofil liefert dagegen sehr sicher die richtige Diagnose [15]. Ein Steroidprofil kann aus der ursprünglichen Blutprobe noch am selben Tag vorliegen. Bedenkt man den oft dramatischen klinischen Verlauf des AGS, so ist der Wert einer unmittelbar korrekten Diagnose augenfällig. Wir empfehlen, die Vorgaben der „Kinder-Richtlinie“ entsprechend zu ändern.

Ähnlich verhält es sich mit der in § 17 „Kinder-Richtlinie“ unter Punkt 12 genannten IVA. Vorgesehen ist die Analytik mit TMS. Da bei dieser Technik eine Auftrennung isobarer Verbindungen vor Eintritt der Probe in das Messgerät nicht erfolgt, werden gleichzeitig mit Isovalerylcarnitin 4 weitere Substanzen erfasst. Negative Ergebnisse schließen eine IVA zwar weitgehend aus, ein positives Messergebnis stellt aber meist einen klinisch irrelevanten Befund dar. Häufig werden falsch-positive Resultate durch eine antibiotische Therapie von Kind oder Mutter mit pivalinsäurehaltigen Antibiotika verursacht. Mit einer ergänzend mit LC-MS/MS durchgeführten Analyse erreicht man dagegen ein korrektes Ergebnis in nahezu 100 % der Fälle [33]. Auch diese *second tier*-Analyse kann in der Regel noch am gleichen Untersuchungstag erledigt werden. Darüber hinaus ist eine sofortige Abklärung noch im Screening-Labor geeignet, wesentliche Folgekosten zu minimieren bzw. zu vermeiden.

Auch die Bestätigung eines positiven Screening-Ergebnisses auf MSUD erfordert eine Abgrenzung von unspezifischen Konzentrationserhöhungen der verzweigten Aminosäuren durch eine Quantifizierung von Alloisoleucin in der Originalblutprobe mit der Technik der LC-MS/MS [34].

### Internationaler Abgleich der Zielkrankheiten

In allen modernen Industriestaaten sind Screening-Programme zur Früherkennung angeborener Krankheiten etabliert. Die Auswahl der Zielkrankheiten zeigt aber große Unterschiede. Dies kann nur teilweise als eine Anpassung an unterschiedliche genetische Hintergründe verstanden werden. Während die „Kinder-Richtlinie“ für Deutschland 16 Zielkrankheiten definiert, umfassen die Programme in Österreich 29, in der Schweiz 10, in England 9, in Frankreich 5 und in Dänemark 19 angeborene Störungen. In den USA listet das Gesundheitsministerium in dem „Recommended Uniform Screening Panel“ [35] sogar 37 *core conditions* plus 26 *secondary conditions* auf.

Medizinische und wissenschaftliche Erkenntnisse, die die Auswertung der in den verschiedenen Ländern laufenden Programme liefert, müssen zwangsläufig zu einer Überprüfung der eigenen Entscheidungen führen.

### Neue Therapien

Durch die Entwicklung neuer Medikamente wie Spiranza® (Nusinersen), Evrysdi® (Risdiplam) und dem Gentherapeutikum Zolgensma® (Onasemnogen-Abeparvovec) für die Behandlung der SMA wurde eine bislang unheilbare Krankheit therapierbar. Dies war der Grund, SMA als Zielkrankheit zu definieren.

Weitere zusätzliche Erkrankungen haben die Chance, bald in das Screening-Programm aufgenommen zu werden. Vermutlich werden bis zum Jahr 2030 mehr als 60 Zell- und Gentherapien von der amerikanischen Food and Drug Agency (FDA) zugelassen [36]. Weitere Erkrankungen sind bereits im Fokus einiger Screening-Programme, teils als Studien, teilweise auch bereits ins Routine-Screening integriert. Hierzu gehören die X‑chromosomale Adrenoleukodystrophie [37], der Adenosin-Desaminase-Mangel (ADA-SCID; [38]), lysosomale Erkrankungen [39] wie der Morbus Pompe [40] und die Mukopolysaccharidosen [41], die nephrotische Cystinose [42] und die metachromatische Leukodystrophie (MLD) [43]. Alle Erkrankungen werden im Anschluss an konventionelle Tests molekulargenetisch gesichert oder unmittelbar mit genetischen Methoden diagnostiziert.

Noch ist es Spekulation, sich wesentlich umfangreichere Programme vorzustellen. In einer Studie in New York City (USA) wird aber immerhin zurzeit bei 100.000 Neugeborenen bereits ein primäres umfassendes genetisches Screening durchgeführt, soweit die Sorgeberechtigten der Studie zustimmen [44]. In Großbritannien plant der National Health Service (NHS) eine ähnliche Studie mit 200.000 Neugeborenen; ca. 200 Zielkrankheiten werden genannt. Es sollen Genvarianten berücksichtigt werden, deren Risiko bekannt ist und die innerhalb der ersten 5 Lebensjahre Symptome hervorrufen. Eltern können auf weitere 100 neurologische Entwicklungsstörungen genetisch untersuchen lassen, welche zwar nicht geheilt werden können, bei denen aber Sprach- und Physiotherapie nachweislich helfen.

Mit solchen Entwicklungen wird man sich auch in Bezug auf das in Deutschland etablierte Screening auseinandersetzen müssen. Vor einer Realisierung müssten allerdings nicht nur medizinische Probleme, sondern auch ethische Fragen und Aspekte des Datenschutzes geklärt werden. Darüber hinaus müssten die Labore auch in die Lage versetzt werden, diese Untersuchungen in großer Zahl innerhalb kurzer Zeit durchzuführen sowie anfallende Datenmengen zu verarbeiten und zu archivieren. Laut einer Umfrage sind viele amerikanische Screening-Zentren überzeugt, dass in Kürze umfangreiche Innovationen erforderlich sind, um das Neugeborenen-Screening auf die rasche Expansion neuartiger Therapien vorzubereiten [45].

## Fazit

Das seit ca. 50 Jahren bestehende Neugeborenen-Screening in Deutschland hat sich in den letzten Jahren rasant weiterentwickelt. Durch den Einsatz neuer Technologien konnte das Untersuchungsspektrum erheblich erweitert werden, um weitere Krankheiten zu erfassen. Die Etablierung von *Second-tier*-Untersuchungen wird das Neugeborenen-Screening zunehmend ergänzen und die Anzahl falsch-positiver Ergebnisse reduzieren. Neue Therapieformen eröffnen die Möglichkeit, weitere Erkrankungen in das Neugeborenen-Screening aufzunehmen und damit eine frühzeitige Behandlung zu ermöglichen. Es ist zu erwarten, dass neben den konventionellen klinisch-chemischen Verfahren zunehmend molekulargenetische Untersuchungen im Rahmen des Neugeborenen-Screenings, aber auch zur gezielten Diagnosesicherung eingesetzt werden.

## Footnotes

1 Zur Verbindlichkeit von Richtlinien, Leitlinien, Empfehlungen und Stellungnahmen siehe https://www.bundesaerztekammer.de/baek/ueber-uns/richtlinien-leitlinien-empfehlungen-und-stellungnahmen. Letzter Zugriff 21.06.2023.
